# Renal heavy and light chain amyloidosis: A rare case report

**DOI:** 10.1097/MD.0000000000042325

**Published:** 2025-04-25

**Authors:** Binbin Chen, Jie Wang, Wenwen Zang, Cong Wang, Jie Li, Ruiheng Yang

**Affiliations:** a Department of Nephrology, Liaocheng People’s Hospital, Liaocheng, Shandong, China; b Department of Pediatric Nephrology, Shandong Provincial Hospital, Jinan, Shandong, China.

**Keywords:** amyloidosis, heavy chain, light chain, renal biopsy

## Abstract

**Rationale:**

Renal heavy and light chain amyloidosis (AHL) is a rare disease primarily diagnosed through renal biopsy. Here, we report a patient with renal AHL. The decision to report this case stems from its rarity. This case provides a reference for the diagnosis and management of this disease.

**Patient concerns:**

A 58-year-old man was admitted to the nephrology department in May 2024 with the chief complaint of lower limb edema over the past year, which had worsened in the preceding 3 months. The clinical presentation included nephrotic syndrome and renal insufficiency.

**Diagnosis:**

The definitive diagnosis was established after comprehensive laboratory tests, bone marrow biopsy, renal pathology, and mass spectrometry analysis.

**Interventions:**

After a definitive diagnosis, the patient was treated with chemotherapy using a combination of bortezomib, dexamethasone, and cyclophosphamide.

**Outcomes:**

The patient’s renal function continued to deteriorate, ultimately leading to hemodialysis treatment.

**Lessons:**

Renal AHL, even though rare, needs to be considered strongly in elderly patients presenting with nephrotic syndrome and renal insufficiency. Cardiac involvement is uncommon. After active treatment, the prognosis is better than that of light chain amyloidosis.

## 1. Introduction

Amyloidosis is a systemic disorder characterized by the abnormal folding and deposition of amyloid proteins in the extracellular matrix, which can affect multiple organs, including the kidneys, liver, heart, soft tissues, and nervous system.^[1]^ To date, 42 precursor substances capable of forming amyloid proteins have been identified,^[2]^ among which heavy and light chain amyloidosis (AHL) is rare. This article presents a case of renal amyloidosis of the AHL type, summarizing its diagnosis, treatment, and prognosis.

## 2. Case presentation

A 58-year-old man was admitted to the nephrology department of our hospital in May 2024, presenting with lower limb edema persisting for over a year and worsening over the past 3 months. The patient had a 10-year history of hypertension. He also had a history of cerebral hemorrhage >10 years ago, resulting in residual left-sided limb weakness. He denied any infectious diseases or family history of hereditary conditions. Physical examination revealed a soft, non-tender abdomen, with no palpable liver or spleen below the costal margin. No percussion pain was noted in either kidney. Significant scrotal edema and severe pitting edema were observed in both lower extremities.

Laboratory examination revealed nephrotic syndrome and renal insufficiency (Table 1). A chest CT scan showed chronic inflammatory changes in both lungs. Ultrasound of the urinary system showed cystic echoes in the right kidney and prostate enlargement. Abdominal ultrasound revealed no abnormalities. Electrocardiogram findings indicated sinus rhythm with mild T-wave changes. Echocardiography revealed no significant abnormalities.

**Table 1:** Laboratory examination on the day of admission.

Items	Values
WBC count	5.47 × 10^9^/L
RBC count	3.05 × 10^12^/L
Hemoglobin	90g/L
Platelet	265 × 10^9^/L
Proteinuria	8343.86 mg/24 h
Albumin	16 g/L
Urea nitrogen	16.37 mmol/L
Creatinine	180.1 umol/L
eGFR	37 mL/min
BNP	75 pg/mL
IgG	2.72 g/L
IgA	1.7 g/L
IgM	0.757 g/L
Serum κ-FLC	44.2 mg/L
Serum λ-FLC	260 mg/L
FLC κ/λ ratio	0.17
Serum PEP	Negative
Urine PEP	Negative

BNP = brain natriuretic peptide, eGFR = estimated glomerular filtration rate, FLC = free light chain, Ig = immunoglobulin, PEP = protein electrophoresis, RBC = red blood cell, WBC = white blood cell, κ = kappa, λ = lambda

**Figure 1.**
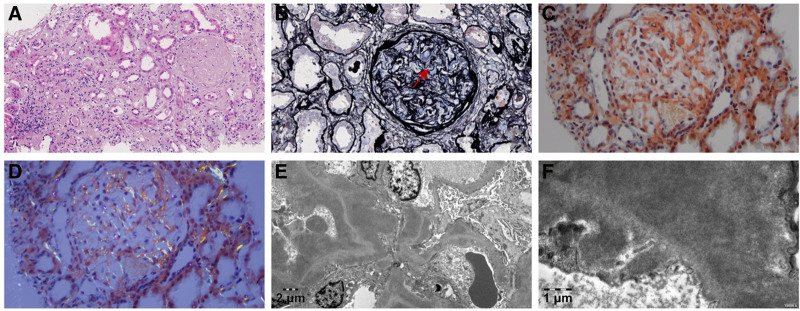
Pathological images of renal biopsy. (A) Deposits of pink, nonstructural material in the glomerular mesangial area, basement membrane, and renal interstitium (HE stain, ×200); (B) segmental “eyelash-like” changes in the basement membrane (PASM stain, ×400); (C, D) Congo red-positive deposits in the glomeruli and renal interstitium, exhibiting apple-green birefringence under polarized light (Congo red stain, ×400); (E, F) Fibrillar material deposition in the mesangium and glomerular basement membrane, characterized by rigidity, lack of branching, and disorganized arrangement (electron microscopy, ×4000, ×15,000). HE = hematoxylin-eosin stain; PASM = periodic acid-silver methenamine stain.

A renal biopsy was performed upon admission. Light microscopy revealed 23 glomeruli. Mild proliferation of mesangial cells and mesangial matrix was noted, with nonstructural widening of the basement membrane and mesangial area. Some glomerular capillary loops were poorly patent, and subepithelial deposits of Congo red-positive proteinaceous material were observed. Renal tubular epithelial cells showed vacuolar and granular degeneration, multifocal loss of brush borders, exposure of basement membranes, multifocal atrophy, and PAS-positive protein casts in the tubular lumen. The renal interstitium showed multifocal lymphocytic and monocytic infiltration with interstitial fibrosis. Small arteries exhibited mild vessel wall thickening, focal nonstructural widening, and no significant luminal narrowing. Congo red staining was positive. Immunofluorescence revealed IgA (−), IgG (++++), IgM (−), tubular (++++), C3 (+), and C1q (−). Electron microscopy demonstrated massive deposition of fibrillar material in the basement membrane and mesangial area, with diameters of approximately 8 to 12 nm. These fibrils were characterized by rigidity, lack of branching, and disordered arrangement (Fig. 1). Based on these findings, the patient was diagnosed with renal amyloidosis.

Bone marrow aspiration revealed that mature plasma cells constituted 1% of the cell population. Mass spectrometry identified high-abundance amyloid-associated proteins in the glomerular region, including apolipoprotein E, apolipoprotein A-IV, and serum amyloid P component, thereby confirming amyloid deposition. Notably, the Igγ profile was most prominent among known typing proteins, with a significant presence of Igλ light chains, suggesting that the amyloid type was AHL (IgG-λ).

The patient was definitively diagnosed with AHL (IgGλ, Mayo 2012 stage II, involving the kidneys). Due to financial constraints, the patient declined treatment with daratumumab and hematopoietic stem cell transplantation. With the consent of the patient and his family, CyBorD (cyclophosphamide, bortezomib, dexamethasone) chemotherapy was initiated. The regimen consisted of bortezomib (2.3 mg on days 1, 4, 8, and 11), cyclophosphamide (400 mg on days 1 and 8), and dexamethasone (20 mg on days 1, 2, 4, 5, 8, 9, 11, and 12). After 1 cycle of chemotherapy, the patient’s renal function progressively worsened with exacerbation of edema, and he developed a severe pulmonary infection. After receiving hemodialysis and anti-infective therapy, the edema resolved, and the pulmonary infection significantly improved. However, the patient declined further chemotherapy and chosed to continue regular hemodialysis therapy.

## 3. Discussion

Renal amyloidosis is a rare disease characterized by the deposition of amyloid proteins in the kidneys. It typically affects middle-aged and elderly individuals, with an insidious onset. Nephrotic syndrome is the primary clinical manifestation and can lead to misdiagnosis or delayed diagnosis. The condition often responds poorly to treatment. Therefore, early diagnosis and treatment are critical for controlling disease progression, improving prognosis, and enhancing survival. Monoclonal-related amyloidosis includes 3 subtypes: light-chain amyloidosis (AL), heavy-chain amyloidosis (AH), and AHL. Among these, AL amyloidosis is the most common, accounting for approximately 90% of amyloidosis cases. The reported incidence of AL amyloidosis in the United States ranges from 6.1 to 10.5 cases per million people.^[3]^ In contrast, AHL amyloidosis is a very rare subtype. The patient, a middle-aged male, presented with nephrotic syndrome and renal insufficiency. A renal biopsy showed Congo red-positive staining in the glomeruli, and electron microscopy revealed extensive deposition of fibrillar material. Mass spectrometry identified the amyloid subtype as AHL (IgG-λ) with renal involvement. Based on these findings, the patient was diagnosed with AHL amyloidosis, a rarely encountered condition in clinical practice.

AHL amyloidosis presents with distinct clinical features compared to AL-type renal amyloidosis. AHL amyloidosis can affect multiple organs, including the kidneys, heart, liver, and gastrointestinal tract, although cardiac involvement is less frequent than in AL-type. Additionally, AHL amyloidosis is associated with a higher likelihood of detecting intact monoclonal immunoglobulins in blood and urine, a higher incidence of hematuria, better overall survival compared to AL-type, and an improved renal response to treatment.^[4]^ Although this patient’s amyloidosis did not involve the heart, a study by Otaka et al found that the heart is the most commonly affected extrarenal organ in AHL.^[5]^ Notably, approximately 50% of patients with AHL amyloidosis have >10% plasma cells in the bone marrow.^[6]^ In our patient, bone marrow cell morphology showed plasma cells comprising <10%, and immunofixation electrophoresis was negative, suggesting a low number of plasma cell clones and low serum M protein content. This emphasizes the importance of considering an AHL diagnosis in clinical practice, even in patients with negative immunofixation electrophoresis results or <10% plasma cells in the bone marrow.

Pathological biopsy of the affected organ or tissue remains the gold standard for diagnosing amyloidosis. Compared to renal biopsy, bone marrow and fat biopsies exhibit lower sensitivity in detecting amyloid proteins.^[4]^ In the kidneys, amyloid deposits typically accumulate in the mesangial area of the glomeruli, the walls of renal arterioles, and the basement membrane. Typical renal amyloid pathology includes mesangial widening without structural changes under light microscopy, capillary loop occlusion, arteriolar wall thickening, and segmental thickening of the basement membrane. Congo red staining is positive, showing apple-green birefringence under polarized light. Electron microscopy reveals disorganized, non-branching fibrils with diameters of 8 to 12 nm. The pathological findings in this patient were consistent with renal amyloidosis. However, in the early stages of the disease, amyloid deposits may be minimal, and the typical pathological features may not be present. As a result, Congo red staining may be negative. Therefore, early renal amyloidosis can be easily misdiagnosed as mesangial proliferative glomerulonephritis, minimal change disease, or membranous nephropathy.^[7]^ Clinically, the diagnosis of early renal amyloidosis primarily relies on electron microscopy of renal tissue, which can detect localized deposits of amyloid fibrils in the mesangial area upon magnification.^[8]^ Following a definitive diagnosis of amyloidosis, further pathological typing is required to determine the specific amyloid subtype. Typing methods include immunohistochemistry, immunofluorescence, immunoelectron microscopy, genetic analysis, and mass spectrometry. Immunofluorescence typing is a commonly used method that utilizes specific antibodies to bind to particular types of amyloid proteins. A fluorescently labeled secondary antibody is then used to detect the location of these antibodies, allowing observation of fluorescent signals under a microscope. This method requires analysis by experienced pathologists. However, we did not use this method due to the unavailability of immunofluorescence reagents at our institution. Immunofluorescence typing of amyloidosis has limitations, particularly in identifying AH and hereditary amyloidosis in routine renal biopsy studies. When immunofluorescence results are inconclusive, other typing methods are required, particularly laser microdissection/mass spectrometry. Laser microdissection/mass spectrometry is a highly accurate test conducted on paraffin sections, capable of distinguishing between AL, AH, AHL, amyloid A renal involvement, and fibrillary glomerulonephritis, with 100% diagnostic specificity and 98% sensitivity.^[9,10]^ In this case, mass spectrometry was employed for typing.

The treatment plan for AHL amyloidosis is primarily based on the approach used for AL-type renal amyloidosis. The primary objectives of treatment are to eliminate plasma cells producing abnormal light and heavy chains, reduce the levels of monoclonal immunoglobulin light and heavy chains in the body, and prevent further amyloid deposition in organs and tissues. The main treatment modalities include hematopoietic stem cell transplantation, bortezomib-based regimens (such as CyBorD), daratumumab combined with CyBorD regimens, immunomodulators, and alkylating agents.^[2]^ Most patients with AHL amyloidosis achieve both hematologic and renal remission following active treatment. Our patient underwent 1 cycle of chemotherapy using the CyBorD regimen. However, deterioration of renal function and a severe infection necessitated the cessation of chemotherapy. During the 3-month follow-up, the patient continued regular hemodialysis. Despite the recommendation for chemotherapy, the patient declined further treatment. Fortunately, the patient’s condition remained stable during this period.

## 4. Conclusion

In conclusion, AHL amyloidosis is a rare condition, and early diagnosis, combined with proactive treatment, can slow disease progression and improve prognosis. This case study serves as a reference for the diagnosis and management of similar patients in the future.

## Acknowledgments

Special thanks to all medical staff of the Department of Nephrology.

## Author contributions

**Conceptualization:** Binbin Chen, Ruiheng Yang.

**Data curation:** Binbin Chen, Jie Wang, Wenwen Zang, Cong Wang, Jie Li.

**Formal analysis:** Jie Wang, Wenwen Zang, Cong Wang, Jie Li.

**Funding acquisition:** Ruiheng Yang.
